# Bleomycin-induced genome structural variations in normal, non-tumor cells

**DOI:** 10.1038/s41598-018-34580-8

**Published:** 2018-11-08

**Authors:** Wilber Quispe-Tintaya, Moonsook Lee, Xiao Dong, Daniel A. Weiser, Jan Vijg, Alexander Y. Maslov

**Affiliations:** Department of Genetics, Albert Einstein College of Medicine, Bronx, NY USA

## Abstract

Many anticancer drugs are genotoxic agents inducing DNA breaks in actively proliferating cancer cells. However, these same drugs also induce mutations, mostly genome structural variations (GSVs). The detection of GSVs in normal cells and tissues is a major challenge due to the very low abundance of these mutations, which are essentially only detectable in clonal outgrowths, such as tumors. Previously we developed Structural Variant Search (SVS) – an NGS-based assay for the quantitative detection of somatic GSVs in normal cells. Using an improved version of SVS we now demonstrate that the same dose of the anti-cancer drug bleomycin induces about 5 times more somatic GSVs in quiescent primary human fibroblasts than in proliferating cells. GVS induction in non-dividing, normal cells was subsequently confirmed *in vivo* by demonstrating that a single dose of bleomycin leads to a significant increase of GSV frequency in mouse liver and heart, two postmitotic tissues. Our findings suggest that normal non-cycling differentiated cells may serve as a reservoir of iatrogenically induced mutations. These results provide more insight into the possible molecular mechanisms that underlie late-life morbidities in cancer survivors exposed to chemotherapy.

## Introduction

Advances in early cancer diagnosis and treatment strategies have resulted in over 14 million cancer survivors in the United States^1^. However, successful treatment is often associated with compromised quality of life due to adverse effects of the applied therapy on normal cells and tissues. Short-term complications, such as hair loss^2^, disorders of excretory^3^ and gastrointestinal^4^ systems, are considered to be a consequence of cytotoxic or cytostatic effects of anticancer drugs on actively dividing host cells and show up shortly after treatment start. Long-term complications of cancer treatment, such as symptoms of premature aging^5^ and secondary neoplasms^6^, are developing years after treatment and their exact mechanism is understudied.

Many anticancer drugs are genotoxic agents and induce apoptosis in actively dividing cancer cells as a response to DNA damage^7^. Notably, virtually all genotoxic anticancer drugs are potent clastogens exerting their cytotoxicity through induction of DNA breaks in affected cells^8^. However, DNA break repair mechanisms (non-homologous end joining and homologous recombination being most prominent^9,10^) are able to recover the DNA chemical structure and rescue the cell, but often at the cost of compromised genome integrity, namely formation of genome structural variants (GSVs)^11^. Hence, it is conceivable that chemotherapy will accelerate the normal age-associated accumulation of GSVs in tissues^12,13^, offering one possible explanation for the multiple late-life morbidities, including secondary malignancies and early onset of symptoms typically associated with aging, in cancer survivors treated with chemotherapy^14–16^.

Chemotherapy-induced GSVs are independent events unique for each affected cell. Thus, while their overall frequency may be high, chemotherapy-induced GSVs involve ultra-low abundant mutations dispersed among affected cells and considered undetectable in normal cells. Thus far, accurate evaluation of these chemotherapy-associated GSVs in normal tissues of cancer patients was not feasible because currently available approaches are not capable of detecting somatic GSVs due to the low abundance of each particular GSV^17^. Recently we developed Structural Variant Search (SVS) – a novel next-generation sequencing (NGS)-based analytical tool for the quantitative detection of somatic GSVs^18^. This assay utilizes ultra-low coverage sequencing data and allows accurate genome-wide assessment of frequency and spectra of low-abundant GSVs induced by bleomycin, a cancer therapeutic agent. Using an improved version of SVS we now show that quiescent cells are significantly more susceptible to the mutagenic effects of chemotherapeutic drug bleomycin than proliferating cells and further demonstrate that GSVs are induced *in vivo* in postmitotic tissues of mice treated with the drug. These results shed light on a possible mechanism of late-life effects of cancer chemotherapy.

## Materials and Methods

### Cell culture and treatment

Human dermal fibroblasts (HDFs) from a 6-year-old male human were provided by H. Choi (Seoul National University). The human fibroblasts were collected and protocols were approved as described in^19^. Cells were maintained in 10% CO_2_ and 3% O_2_ atmosphere at 37 °C in DMEM (Gibco, Grand Island, NY, USA) supplemented with 10% FBS (Gibco). The quiescent state of the cells was achieved by culturing in the medium supplemented with 0.1% of FBS for 24 hours prior to the experiment start. For the treatment serum-free medium with bleomycin (Calbiochem, San Diego, CA, USA) was prepared at the time of application from stock solution of the drug (10 mM in DMSO) and applied for 1 hour at the atmospheric O_2_ level. At the end of the treatment, cells were washed twice with PBS and cultured further in the medium supplemented either with 10% FBS (proliferating cells) or 0.1% FBS (quiescent cells). Proliferating cells were trypsinized 24 hours and 72 hours after the treatment, half of the cells was collected for DNA extraction and half was left in culture until the next time point. Quiescent cells were maintained without passaging and collected for DNA extraction at the time points designated in the text.

### Cell counting and viability assessment

To determine viable cell numbers, trypsinized cells were stained using Guava ViaCount Reagent (MilliporeSigma, Burlington, MA, USA) and analyzed using Guava EasyCyte Plus flow cytometer (MilliporeSigma) as recommended by the manufacturer. The cell doubling time was calculated using the formula:

$$Doubling\,Time=Time\,Period\ast log({2})/[log(Final\,Conc)-log(Initial\,Conc)]$$

### Animals and tissue collection

All procedures involving animals were approved by the Institutional Animal Care and Use Committee (IACUC) of Albert Einstein College of Medicine and performed in accordance with relevant guidelines and regulations. Six three-month-old female C57BL/6 mice were obtained from the National Institute on Aging and maintained in the animal husbandry facility of the Albert Einstein College of Medicine until treatment. A single dose of bleomycin in saline (40 mg/m^2^) was delivered by IP injection; control mice received an injection of saline. Mice were euthanized 24 hours after injections and liver, lung and heart tissues were collected aseptically and kept at −80 °C until use.

### DNA isolation and quantification

Total DNA was isolated from tissue and cultured cells using the Quick-gDNA^TM^ MiniPrep kit (Zymo Research Corporation, Irvine, CA, USA) following the manufacturer’s instructions. DNA concentration was determined using the Qubit 2.0 fluorimeter (Life Technologies Corporation, Carlsbad, CA, USA).

### Long-range PCR and preparation of a chimera-free “artificial genome”

Long-range PCR was performed with the Takara LA PCR kit (Takara Bio Inc., Otsu, Shiga, Japan) according to the manufacturer's protocol. Five DNA fragments were amplified from the mouse genomic DNA using previously designed and validated primers targeting distant regions of the mouse genome (Table 1)^20^.

**Table 1:** Primer pairs used for long-range PCR.

Fragment name	Genome region	Primer pair	Product size (bp)
Alb	chr5:90462646–90472576	5′-GCTGGTTGGGAGAGCCACTT-3′ 5′-AGAGCAGAGAAGCATGGCCG-3′	9931
Ptprz1	chr6:23000097–23009978	5′-ACCGAAGTGACACCACAGGC-3′ 5′-GCACACCTCCCTACCTGCTC-3′	9882
Ercc1	chr7:19342571–19352554	5′-AACTCAAAGCCCCCGAGTGG-3′ 5′-GCTGGGGAGAGAGACAGCAC-3′	9984
Gabra1	chr11:42135420–42145769	5′-CACGCTTTTGCCATCCCACG-3′ 5′-CTCTGCCCTCAGCTTTGCCT-3′	10350
Faim2	chr15:99501793–99512177	5′-ACTGAGAACCCCGGGAGGAT-3′ 5′-TCCTGGGGGACCAGCTAAGT-3′	10385

Obtained amplicons were mixed together in equimolar quantities and used as a template for sequencing library preparation. The resulting “artificial genome” is guaranteed free from “interchromosomal” rearrangements between these five fragments simulating different chromosomes because each of them was amplified individually and chances that potential PCR-mediated errors will produce rearrangements between these particular regions of genome are negligibly small. Possible PCR-mediated recombination between target DNA fragments and the rest of the genome occurring via template-switching mechanism^21^ were filtered out during analysis of the potential variants.

### SVS assay

The SVS method used has been described previously^18^, but some modifications were made to improve its accuracy further. The improvements mainly reside in sequencing libraries preparation protocol. Ligation-based sequencing libraries were prepared using NEBNext Fast DNA Fragmentation & Library Prep Set for Ion Torrent (NEB, Ipswich, MA, USA), following the manufacturer’s instructions. Transposon-based libraries were prepared as we described previously^22^. Ligation-mediated chimera-free (LCF) libraries were prepared as follows:

1. Fragmentation using NEBNext dsDNA Fragmentase (NEB);
2. End-repair I using S1 nuclease (Thermo Fisher Scientific, Waltham, MA USA);
3. A-tailing using Terminal Transferase (TdT) polymerase (NEB) and dATP (NEB);
4. Ligation of single-stranded sequencing adaptors using Taq DNA Ligase (NEB). The sequencing adaptor oligos were: 5′-CCTCTCTATGGGCAGTCGGTGATTTTTTTT-3′ (universal adaptor) and 5′-CCATCTCATCCCTGCGTGTCTCCGACTCAG**NNNNNNNNNN**TTTTTTTT-3′ (barcoded adaptors, where NNNNNNNNNN represents an IonXpress barcode);
5. End-repair II using T4 DNA polymerase (NEB) and dNTP mix (NEB).

All completed libraries were size selected using the PippinHT System (Sage Science, Beverly, MA, USA) and quantified using KAPA Library Quantification Kit for Ion Torrent Platform (Kapa Biosystems, Inc., Wilmington, MA, USA). Sequencing was performed on Ion Proton System (Life Technologies Corporation) using the Ion PI™ Hi-Q Sequencing 200 Kit and Ion PI™ Chip Kit v3, following the manufacturer’s instructions. Sequencing data analysis was performed using our proprietary software as described previously^18^.

### Statistical analysis

The descriptive statistics and the P values were calculated using a two-sample t-test (Microsoft Excel software package).

## Results

### Optimization of SVS assay

**Figure 1:**
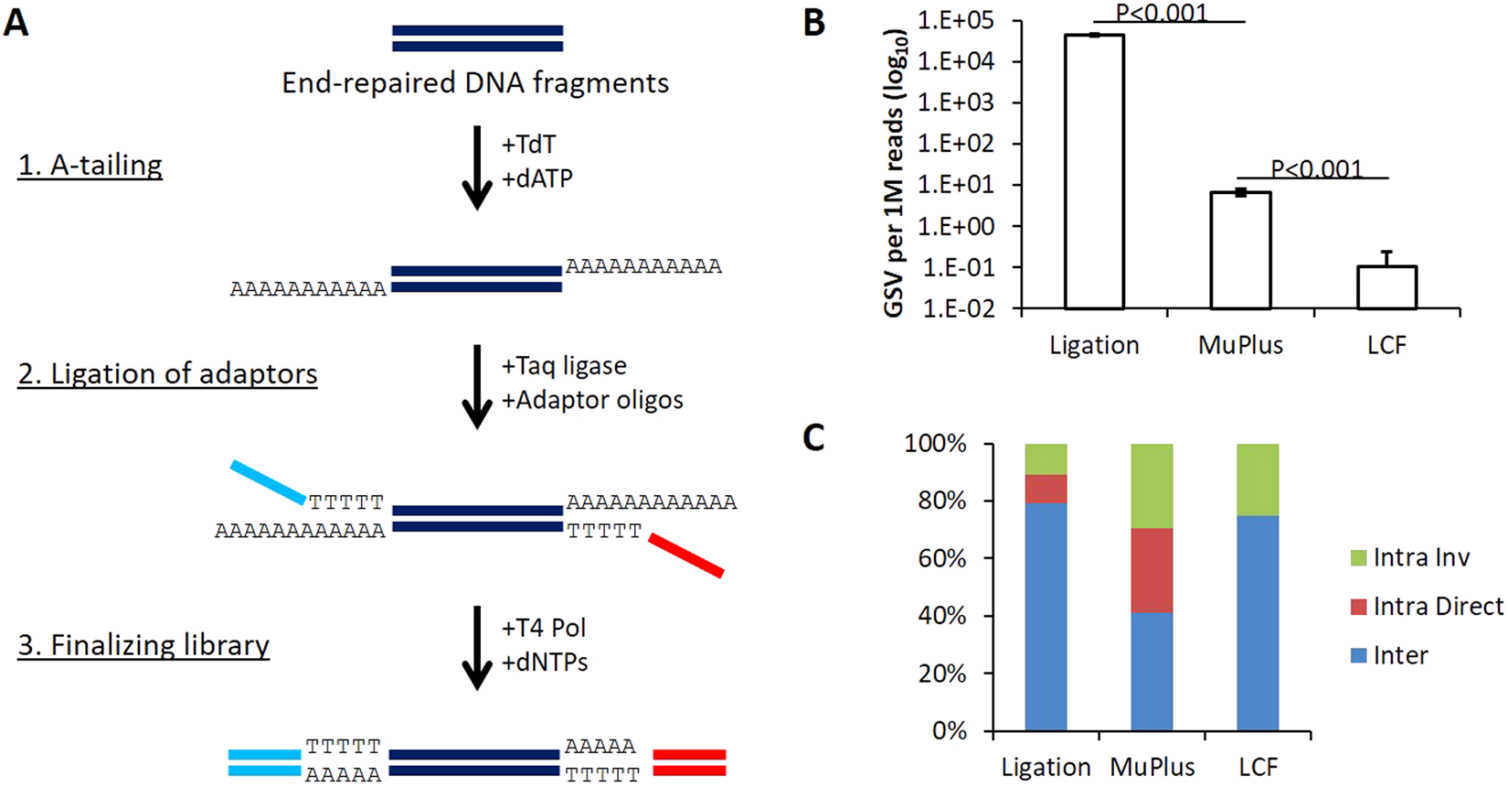
Ligation-mediated Chimera-Free (LCF) protocol ensures preparation of sequencing libraries virtually free from artificial chimeras. (**A**) LCF protocol outline. LCF is based on assignment of sequencing adapters as single-stranded oligonucleotides at elevated temperature using thermostable Taq DNA ligase. The ligation is facilitated by hybridization of adapter carrying thymine residuals on 3′-end and DNA fragment with A-overhangs at 3′-ends of both strands. At the final step sequencing library is completed by treatment with T4 DNA polymerase in the presence of dNTPs. (**B**) Frequency of artificial chimeras in sequencing libraries prepared with different approaches. (**C**) Spectra of artificial chimeras in sequencing libraries prepared with different approaches. Data in (**B**) shown as the average ± s.d.; n = 3 for each, ligation-based and MuPlus libraries and n = 8 for LCF libraries; statistically significant differences determined by two-tailed t-test.

Sequencing libraries free from artificial chimeric fragments are essential for accurate detection of somatic low-abundant GSVs using SVS assay^18^. To improve accuracy of GSV calling in SVS we have designed a Ligation-mediated Chimera-Free (LCF) sequencing library preparation protocol (Fig. 1A). To validate our new protocol, we have compared sequencing results of LCF libraries with that of libraries created using conventional ligation-based PCR-free approach and transposon-based MuPlus libraries^22^. As a template DNA for libraries preparation we used a chimera-free “artificial genome”. We found that frequency of artifacts per 1 million sequencing reads in transposon-based libraries is more than three orders of magnitude lower than in libraries prepared with a ligation-based approach (6.73 and 43,003.08 respectively). Utilization of the PCR-free LCF protocol resulted in further suppression frequency of artifacts to 0.08 rearrangements per 1 million sequencing reads (Fig. 1B). Further analysis of identified artificial rearrangements revealed that a majority (~80%) of artifacts in the ligation-based PCR-free libraries were interchromosomal rearrangements; the remainder were intrachromosomal rearrangements with equal representation of artifacts composed of DNA fragments with direct or opposite (inversions) orientations relative to the reference genome. Transposon-based libraries contained approximately equal fractions of these three types of rearrangements with slight prevalence of interchromosomal rearrangements. Analysis of more than 50 million reads obtained from the libraries prepared with the LCF protocol revealed four rearrangements – three interchromosomal rearrangements and one intrachromosomal inversion (Fig. 1C and Supplementary Table 1). Thus, the LCF protocol allows construction of sequencing libraries virtually free from chimeric DNA fragments.

### Bleomycin-induced GSVs in proliferating and quiescent human normal primary cells

**Figure 2:**
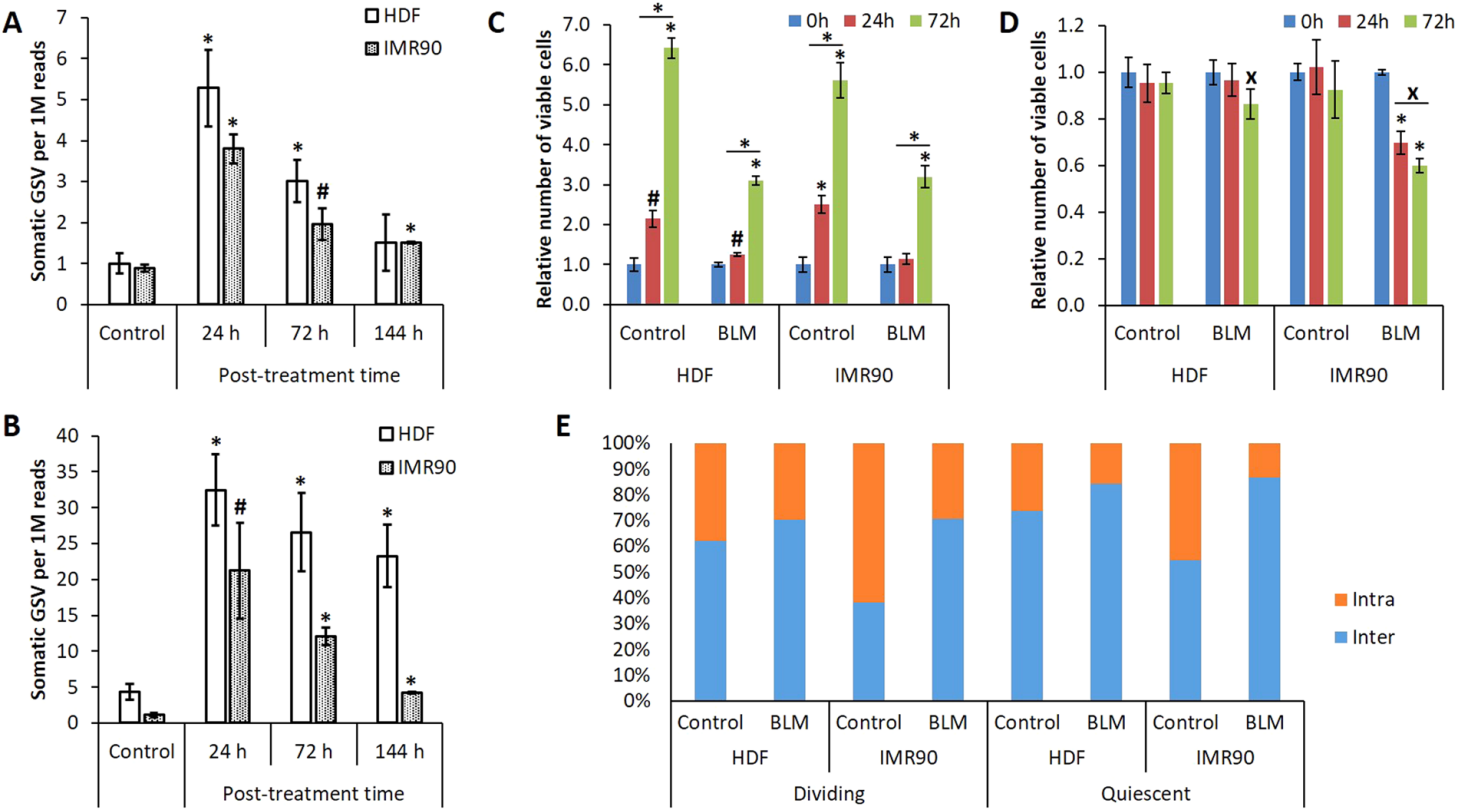
Quiescent human primary cells are more susceptible to the mutagenic effects of bleomycin. (**A**) Somatic GSV frequency in actively proliferating HDFs and IMR90 cells at different time points after treatment with bleomycin. (**B**) Somatic GSV frequency in quiescent HDFs and IMR90 cells at different time points after treatment with bleomycin. (**C**) Relative amount of proliferating viable cells at different time points after treatment with bleomycin. (**D**) Relative amount of quiescent viable cells at different time points after treatment with bleomycin. (**E**) Spectra of somatic GSVs in actively proliferating and quiescent HDFs and IMR90 cells after treatment with bleomycin (24 hours time point). Data in (**A**–**D**) shown as the average ± s.d.; for each data point n = 5 for HDFs and n = 3 for IMR90 cells; statistically significant differences versus corresponding controls and adjacent time points determined by two-tailed t-test [^x^p < 0.05, ^#^p < 0.01, *p < 0.001].

After optimizing the assay, we applied SVS to assess the frequency of somatic GSVs induced by the antineoplastic drug bleomycin in proliferating human dermal fibroblasts (HDFs) and human lung fibroblasts (IMR90) at different time points after treatment. As expected, bleomycin treatment led to a statistically significant increase of somatic GSV frequency in both cell strains with the maximum observed value at 24 hours after treatment (Fig. 2A and Supplementary Table 2). Further monitoring revealed that drug-induced GSV load in proliferating HDF and IMR90 cultures was progressively declining – the GSV frequency was about two thirds at 72 hours post-treatment and less than half of the maximum values 144 hours after the treatment. Of note, the difference in GSV frequency between control HDFs and HDFs treated with bleomycin was no longer statistically significant by 144 hours after treatment (Fig. 2A).

Next we analyzed GSV frequency induced by bleomycin treatment in quiescent HDFs and IMR90 cells. The quiescence state was generated by culturing cells at low serum concentration (0.1% FBS). Similar to its effect on proliferating cells, bleomycin caused a statistically significant increase of GSVs in quiescent cells of both strains at 24 hours after treatment (Fig. 2B and Supplementary Table 2). However, the mutagenic response of quiescent cells was significantly more prominent than that of proliferating cells (32.5/21.2 versus 5.3/3.8 GSVs per one million sequencing reads for HDFs/IMR90, respectively). Interestingly, further kinetics of the bleomycin-induced GSVs was different in the two different cell strains. The level of GSVs in quiescent HDFs remained relatively stable over a period of 144 hours. Although there was a trend of decline this was not statistically significant (Fig. 2B). By contrast, quiescent IMR90 cells demonstrated rapid loss of induced GSVs (Fig. 2B), similar to that observed in proliferating cells.

### Growth and survival of proliferating and quiescent human normal primary cells after bleomycin treatment

Not unexpectedly, bleomycin treatment of actively dividing human cells led to a significant decrease in cell growth rate during the first 24 hours after the intervention; population doubling time was ~75 and ~124 hours compared to ~22 and 18 hours for control HDFs and IMR90 cells, respectively (Fig. 2C). However, during the following 48 hours (72 hours post-treatment) the growth rate of treated cells was almost completely restored (population doubling time 36 and 32 hours for treated HDF and IMR90 cells, respectively). Treatment with bleomycin virtually did not affect viability of quiescent HDFs in the first 24 hours after exposure to the drug, but led to slight decrease in viable cell number in the following 48 hours (p < 0.05). In contrast, quiescent IMR90 cells lost ~30% of the population during the first 24 hours after the treatment (p < 0.001) and cells continued to die during the following 48 hours (Fig. 2D). Notably, both, HDF and IMR90 control populations remained stable during the course of the experiment, confirming quiescent state of the cells (Fig. 2D).

### Spectra of bleomycin induced GSVs in proliferating and quiescent human normal primary cells

Treatment with bleomycin caused substantial changes in the type of somatic GSVs in proliferating HDFs and IMR90 cells (Fig. 2E). While frequencies of both inter- and intrachromosomal rearrangements were increased 24 hours after the treatment, GSVs induced by bleomycin treatment in proliferating cells were mostly interchromosomal GSVs. This effect was more prominent in IMR90 cells where the fraction of interchromosomal GSVs grew from 38% to 70%. The relative representation of intrachromosomal rearrangements declined in both cell strains, most noticeable in proliferating IMR90 cells (61% in control cells vs. 29% in treated cells). Similar to proliferating cells bleomycin-treated quiescent HDFs and IMR90 cells had an increased fraction of interchromosomal rearrangements in comparison with control and a reduced fraction of intrachromosomal rearrangements, also in comparison with control cells. As in proliferating cells these changes in the mutational spectra were more prominent in IMR90 cells. Thus, mutation spectra of bleomycin-induced GSVs are similar in actively dividing and quiescent cells.

### Susceptibility of mouse tissues to the mutagenic effects of bleomycin

**Figure 3:**
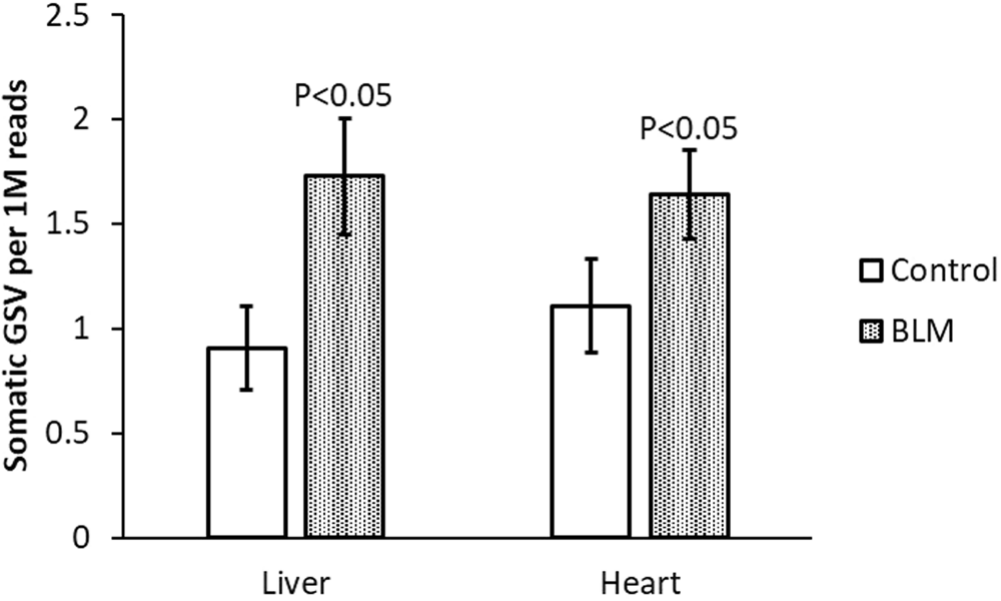
Frequency of GSVs in mouse tissues at 24 hours after single bleomycin (BLM) dose. Data shown as the average ± s.d.; n = 3 for each data point; statistically significant differences versus corresponding controls determined by two-tailed t-test.

Our observation that bleomycin readily induces GSVs in non-dividing cells prompted us to directly test the possible induction of these mutations in mammalian postmitotic tissues *in vivo*. For this purpose we used C57BL/6 mice treated with sub-lethal doses of bleomycin. The experimental group received an intraperitoneal (IP) injection with bleomycin (40 mg/m^2^) in saline, while control animals were injected with the same (0.5 ml) volume of saline only. Mice were sacrificed for analysis 24 hours after the treatment. We found that a single IP injection of bleomycin induced a statistically significant elevation of somatic GSV frequency in the mouse liver and heart tissues (Fig. 3 and Supplementary Table 3). These results demonstrate that bleomycin readily induces GSVs *in vivo* in postmitotic tissue.

## Discussion

Detection of somatic mutations using next-generation sequencing (NGS) is a challenging task due to their low abundance, which makes them indistinguishable from NGS-associated errors. Several approaches have been introduced allowing assessment of point mutations and small indels, but not genome structural variants (GSVs)^17^. Recently we have addressed this problem by developing SVS, the first practical assay for quantitative detection of low abundant GSVs^18^. The main feature of SVS is utilization of a sequencing library depleted from artificially created chimeric sequences. This was achieved by employment of a transposon-based library preparation protocol lacking a ligation step, the main source of artifacts^22^. We previously demonstrated that this provided high enough sensitivity and specificity to detect a dose response relationship of GSVs after treatment of cells with bleomycin and etoposide^18^. Here we used the same approach combined with a newly developed library preparation protocol to achieve a further increase in accuracy by omitting the PCR amplification step.

Using bleomycin-treated quiescent cells as a surrogate for non-dividing cells in cancer patients treated with chemotherapeutic drugs we found that cells withdrawn from cycling are susceptible to bleomycin-induced mutagenesis even more than actively proliferating cells. Notably, this phenomenon was observed with two different normal primary human cells, confirming that the observed effect does not depend on genetic features of the cells, but rather has general character. Of note, these results are in line with previously reported increased accumulation of genome rearrangements in quiescent cells upon treatment with hydrogen peroxide^23^. One plausible explanation of the observed more than 5-fold difference in mutational response is that dividing and quiescent cells cope with DNA DSBs in a different way. While bleomycin-induced DNA DSBs in proliferating cells are predominantly repaired by relatively error-free homologous recombination mechanism, the repair of DNA DSBs in quiescent cells is mediated by error-prone non-homologous end joining, resulting in significantly higher amounts of somatic GSVs^24,25^.

DNA mutations are stable irreversible changes in DNA sequence and the observed decline of somatic GSV load in actively proliferating cells was not expected because cells can repair DNA damage such as breaks, but lack mechanisms capable of restoring sequence integrity. Hence, once mutations are generated, as a consequence or erroneous repair or replication, they should be irreversible and remain in the affected cell population unless cell death removes a mutated cell. Thus, the most likely explanation of the decrease of GSVs in proliferating cells is a growth advantage of cells with low levels of GSVs. Heavily mutated cells would either die or display a significantly lower growth rate. In our experiment with proliferating cells treated with bleomycin, the cells with the lowest mutation load, continue to proliferate and dilute the cells with higher numbers of mutations. Indeed, unlike proliferating cell populations where both HDFs and IMR90 cells were rapidly losing bleomycin-induced GSVs after 24 hours, quiescent HDFs, least susceptible to the toxic effects of the drug of the two cell strains used (Fig. 2D), showed only a very small decline. While mutational loads in HDFs remained relatively stable over the period of the experiment, the quiescent IMR90 cells did show a significant decline in GSV frequency, similar to proliferating ones. This is most likely due to the higher toxicity of bleomycin in IMR90, in which significant cell death was noticed upon treatment with bleomycin (Fig. 2D). We assume that genetic differences between the two primary human fibroblast strains are responsible for these different outcomes. This observation emphasizes the importance of taking in consideration individual genetic features of the patients subjected to chemotherapy and needs further investigation.

Our subsequent *in vivo* experiments confirm our observations with quiescent cells in culture. They demonstrated that a single dose of bleomycin leads to a statistically significant increase of somatic GSV frequency in the mouse liver and heart, two postmitotic tissues.

In summary, using our new, NGS-based method for direct GSV detection in normal, non-clonally developed tissue we provide clear evidence indicating that non-cycling differentiated cells can serve as a reservoir of iatrogenically induced somatic mutations. Our present findings provide an immediate approach for studying the molecular mechanisms of late morbidities in cancer survivors exposed to chemotherapy. This is particularly important for childhood cancer survivors whose successful treatment is often associated with compromised quality of consecutive life, which averages more than 60 years^26^. SVS provides a practical approach for further systematic studies addressing an urgent unmet need to understand and predict how chemotherapy affects normal tissue and leads to adverse events.

## Electronic supplementary material

Supplementary tables
