# Supplementary material for: Bleomycin-induced genome structural variations in normal, non-tumor cells
Source: Sci Rep. 2018 Nov 8;8:16523. doi: 10.1038/s41598-018-34580-8 (PMC6224559; doi:10.1038/s41598-018-34580-8)
Supplement: Supplementary file 1 — Supplementary tables [file 41598_2018_34580_MOESM1_ESM.pdf]

Bleomycin-induced genome structural variations in normal, non-tumor cells

Wilber Quispe-Tintaya[1], Moonsook Lee[1], Xiao Dong[1], Daniel A. Weiser[1], Jan Vijg[1*], Alexander Y. Maslov[1*]

[1]Department of Genetics

Albert Einstein College of Medicine

Bronx, NY, USA.

Supplementary Table 1. Sequencing statistics and number of detected artificial rearrangements in sequencing libraries prepared with different approaches.

#	Library preparation protocol	Total number of sequencing reads	Total bases	Average read length	Rearrangements detected			
					Interchromosomal	Intrachromosomal direct	Intrachromosomal inverted	Total
1	Ligation	4,870,314	8.82E+08	181	168,296	20,568	22,528	211,392
2	Ligation	4,903,572	8.95E+08	182	176,530	22,153	24,469	223,152
3	Ligation	5,164,676	9.31E+08	180	163,912	20,885	22,291	207,088
4	MuPlus	16,591,299	3.07E+09	184	51	24	29	104
5	MuPlus	12,454,296	2.28E+09	183	28	21	21	70
6	MuPlus	10,848,413	1.99E+09	183	30	32	28	90
7	LCF	3,301,616	6.49E+08	196	0	0	1	1
8	LCF	3,210,002	6.53E+08	203	0	0	0	0
9	LCF	4,586,136	9.19E+08	200	0	0	0	0
10	LCF	9,325,107	1.83E+09	196	1	0	0	1
11	LCF	9,557,736	1.88E+09	196	1	0	0	1
12	LCF	4,801,042	9.61E+08	200	0	0	0	0
13	LCF	9,243,208	1.8E+09	194	0	0	0	0
14	LCF	6,538,104	1.26E+09	193	1	0	0	1

Supplementary Table 2. Sequencing statistics and number of detected somatic GSVs in human dermal fibroblasts.

Cell strain	Proliferation status	Treatment	Total number of sequencing reads	Total bases	Average read length	Rearrangements detected			
						Interchromosomal	Intrachromosomal direct	Intrachromosomal inverted	Total
HDF	Dividing	Control	6,147,661	1.21E+09	196	1	3	0	4
			6,179,847	1.22E+09	197	4	2	0	6
			5,031,399	9.97E+08	198	2	2	1	5
			9,344,154	1.93E+09	206	8	2	0	10
			9,001,915	1.77E+09	196	8	3	1	12
		BLM, 24h	4,921,433	9.54E+08	193	11	4	7	22
			5,974,759	1.18E+09	198	19	4	4	27
			8,115,001	1.6E+09	197	24	11	5	40
			4,905,485	1.02E+09	207	23	3	3	29
			5,473,137	1.08E+09	197	31	3	2	36
		BLM, 72h	6,541,396	1.26E+09	192	10	1	3	14
			5,512,212	1.1E+09	199	16	1	0	17
			22,131,635	4.27E+09	193	43	21	5	69
			4,985,544	9.77E+08	195	6	7	3	16
			7,385,685	1.48E+09	199	19	3	4	26
		BLM, 144h	2,858,430	5.49E+08	192	1	1	1	3
			6,502,975	1.3E+09	200	4	3	0	7
			5,668,222	1.11E+09	195	2	3	2	7
			7,262,142	1.45E+09	199	7	2	2	11
			6,313,110	1.27E+09	201	13	2	2	17
	Quiescent	Control	5,729,781	1.12E+09	195	14	3	0	17
			9,540,064	1.87E+09	195	24	5	2	31
			6,288,108	1.22E+09	193	21	7	3	31
			3,248,430	6.49E+08	199	12	3	2	17
			6,426,921	1.26E+09	196	25	8	1	34
		BLM, 24h	7,717,135	1.47E+09	190	167	22	18	207
			5,058,931	1.03E+09	202	137	11	9	157

			6,513,153	1.3E+09	200	175	24	8	207
			2,874,352	5.65E+08	196	76	14	3	93
			6,017,566	1.24E+09	205	210	25	8	243
		**BLM, 72h**	6,266,749	1.2E+09	190	111	10	8	129
			5,202,098	1.09E+09	209	108	15	3	126
			3,260,598	6.57E+08	201	76	8	0	84
			4,023,852	8.04E+08	199	89	15	4	108
			6,529,173	1.3E+09	198	201	19	11	231
		**BLM, 144h**	2,906,356	5.9E+08	203	43	6	4	53
			7,195,328	1.42E+09	196	113	20	6	139
			6,910,988	1.34E+09	194	134	18	13	165
			2,777,736	5.7E+08	205	57	10	8	75
			9,570,740	1.84E+09	192	229	27	10	266
**IMR90**	**Dividing**	**Control**	10,149,497	2.1E+09	207	2	6	0	8
			7,506,249	1.51E+09	200	4	3	0	7
			6,378,313	1.3E+09	203	2	3	1	6
		**BLM, 24h**	7,285,818	1.41E+09	193	19	6	0	25
			6,291,777	1.19E+09	189	17	6	1	24
			6,268,300	1.23E+09	196	17	5	4	26
		**BLM, 72h**	7,416,915	1.44E+09	193	8	4	0	12
			6,327,077	1.27E+09	200	6	3	3	12
			6,733,012	1.35E+09	200	8	3	5	16
		**BLM, 144h**	6,633,386	1.31E+09	197	4	5	1	10
			7,290,640	1.45E+09	198	7	4	0	11
			7,124,755	1.41E+09	198	7	3	1	11
	**Quiescent**	**Control**	6,435,414	1.26E+09	195	4	1	1	6
			5,858,887	1.17E+09	199	4	0	2	6
			6,641,877	1.33E+09	200	4	5	1	10
		**BLM, 24h**	6,134,504	1.23E+09	199	72	11	1	84
			6,492,888	1.26E+09	193	133	16	4	153
			6,851,075	1.33E+09	193	157	17	7	181
		**BLM, 72h**	6,181,868	1.2E+09	194	44	19	3	66
			6,517,560	1.27E+09	194	60	18	3	81

			6,304,231	1.22E+09	194	66	11	5	82
		**BLM, 144h**	5,934,413	1.14E+09	192	19	3	2	24
			5,889,351	1.13E+09	192	20	5	0	25
			6,318,817	1.22E+09	193	18	6	3	27

Supplementary Table 3. Sequencing statistics and number of detected somatic GSVs in mouse tissues.

Treatment	Tissue	Total number of sequencing reads	Total bases	Average read length	Rearrangements detected			
					Interchromosomal	Intrachromosomal direct	Intrachromosomal inverted	Total
Control	Heart	6,498,294	1.27E+09	195	4	0	2	6
		7,626,367	1.5E+09	197	6	1	1	8
		8,874,074	1.74E+09	196	6	1	5	12
	Liver	4,372,769	8.81E+08	202	0	0	3	3
		8,422,078	1.65E+09	196	2	3	3	8
		8,362,078	1.67E+09	199	6	2	1	9
BLM	Heart	5,334,878	1.04E+09	196	6	0	2	8
		7,161,123	1.41E+09	196	7	3	1	11
		5,826,925	1.16E+09	199	8	2	1	11
	Liver	6,834,133	1.34E+09	196	4	3	3	10
		7,046,433	1.38E+09	195	4	2	6	12
		8,449,269	1.68E+09	199	10	5	2	17
